# Reading Derived Words by Italian Children With and Without Dyslexia: The Effect of Root Length

**DOI:** 10.3389/fpsyg.2018.00647

**Published:** 2018-05-08

**Authors:** Cristina Burani, Stefania Marcolini, Daniela Traficante, Pierluigi Zoccolotti

**Affiliations:** ^1^Istituto di Scienze e Tecnologie della Cognizione – Consiglio Nazionale delle Ricerche, Rome, Italy; ^2^Department of Life Sciences, University of Trieste, Trieste, Italy; ^3^Department of Psychology, Università Cattolica del Sacro Cuore, Milan, Italy; ^4^Dipartimento di Psicologia, Sapienza Università di Roma, Rome, Italy; ^5^Neuropsychology Unit, Fondazione Santa Lucia (IRCCS), Rome, Italy

**Keywords:** word length, reading, orthographic depth, transparent orthography, children, dyslexia, morphology, root length

## Abstract

Children with dyslexia are extremely slow at reading long words but they are faster with stimuli composed of roots and derivational suffixes (e.g., CASSIERE, ‘cashier’) than stimuli not decomposable in morphemes (e.g., CAMMELLO, ‘camel’). The present study assessed whether root length modulates children’s morphological processing. For typically developing readers, root activation was expected to be higher for longer than shorter roots because longer roots are more informative access units than shorter ones. By contrast, readers with dyslexia were not expected to be facilitated by longer roots because these roots might exceed dyslexics’ processing capacities. Two groups of Italian 6th graders, with and without dyslexia, read aloud low-frequency derived words, with familiar roots and suffixes. Word reaction times (RTs) and mispronunciations were recorded. Linear mixed-effects regression analyses on RTs showed the inhibitory effect of word length and the facilitating effect of root frequency for both children with and without dyslexia. Root length predicted RTs of typically developing readers only, with faster RTs for longer roots, over and above the inhibitory effect of word length. Furthermore, typically developing children had faster RTs on words with more frequent suffixes while children with dyslexia were faster when roots had a small family size. Generalized linear regression analyses on accuracy showed facilitating effects of word frequency and suffix frequency, for both groups. The large word length effect on latencies confirmed laborious whole-word processing in children when reading low-frequency derived words. The absence of a word frequency effect along with the facilitating effect of root frequency indicated morphemic processing in all readers. The reversed root length effect in typically developing readers pointed to a stronger activation for longer roots in keeping with the idea that these represent particularly informative units for word decoding. For readers with dyslexia the facilitating effect of root frequency (not modulated by root length) confirmed a pervasive benefit of root activation while the lack of root length modulation indicated that the longest roots were for them too large units to be processed within a single fixation.

## Introduction

In languages with transparent orthography, like Italian, reading through grapheme-to-phoneme conversion rules leads to accuracy levels almost as high as reading through access to lexical representations, but it may be more time consuming. Most Italian children with developmental dyslexia show an extremely slow and analytical reading behavior (Zoccolotti et al., 1999, 2005), which is probably due to a massive use of the extra-lexical route. They typically make several small amplitude saccades accompanied by long-lasting fixations within a word (De Luca et al., 1999, 2002; see also Hutzler and Wimmer, 2004). They usually read rather accurately, but very slowly and serially (Spinelli et al., 2005). Within the psycholinguistic grain size theory proposed by Ziegler and Goswami (2005), this reading behavior can be seen as a failure in developing reading units of a large grain size (i.e., words, Hawelka et al., 2010), possibly because of limitations in their visuo-perceptual processing (e.g., Bosse et al., 2007; Martelli et al., 2009).

As a consequence, children with dyslexia typically experience great difficulties in reading long stimuli. However, long words that contain morphemes (roots and affixes) are read aloud by them faster than matched words not composed of morphemes. In several studies (for a review, see Burani, 2010) we showed that word naming times of children with dyslexia were shorter for stimuli composed of a root and a derivational suffix (e.g., CASS-IERE, ‘cashier’), as compared to simple words of the same length and frequency not parsable in root + derivational suffix (e.g., CAMMELLO, ‘camel’; Burani et al., 2008). We proposed that children with limited reading ability may find morphemes useful because morphemes are reading units of an intermediate grain size with respect to graphemes on the one side and words on the other: Morphemes are larger reading units than single graphemes (which entail slow analytical sub-lexical processing) but at the same time they are shorter reading units as compared to the word, which is too long for them to be processed in a single fixation as a whole. As a consequence of their formal and lexical characteristics, morphemes can be exploited to increase reading fluency (see also Deacon et al., 2016).

A facilitation on reading times due to the morphological composition of the stimulus was also found in typically developing readers at different ages. However, whereas skilled readers were facilitated by morphemes only when they were present in newly encountered words (i.e., pseudowords; Burani et al., 2002) and in low-frequency words (see also Carlisle and Stone, 2005; Deacon et al., 2011), children with dyslexia were facilitated by the presence of morphemes both in reading new words and words of various frequencies, including high-frequency words (Burani et al., 2008; Marcolini et al., 2011). Overall, the facilitating effect of the word’s morphological composition was larger in children with dyslexia as compared to skilled readers of the same age (see also Elbrö and Arnbak, 1996; Carlisle and Stone, 2005; Suárez-Coalla and Cuetos, 2013).

For both children with dyslexia and skilled readers, the facilitating effect on vocal reaction times (RT) to pseudowords was mainly driven by the root, not the suffix (Traficante et al., 2011). This finding was interpreted as the combined effect of the main lexical role of the root which provides a head-start to morphemic decomposition (Bertram and Hyönä, 2003) and the serial reading behavior which is typical of developing readers of a transparent orthography.

Several properties contribute to the leading role of the root in morpheme-based reading. A number of studies reported effects of base frequency on English-speaking children’s reading of derived words. Mann and Singson (2003) found that third- and sixth-grade children were more accurate in reading derived words with high- than with low-base frequencies. Similar effects were reported by Carlisle and Stone (2005) on grade 4 and 5 (but not on grade 2 and 3) children. Deacon et al. (2011) replicated these findings and extended them to the reading speed of children in grades 4, 6, and 8. In both the latter studies, morphemic effects were apparent on low frequency derived words. Carlisle and Katz (2006) also showed that grade 4 and 6 English-speaking children read derived words with large and frequent morphological families (i.e., large family size and high family frequency) more accurately than words with small and less frequent morphological families.

While the effect of frequency of the root on the processing of morphological words has been attested, it is much less known which other factors may contribute in modulating the influence of the root. Laudanna and Burani (1995) have proposed that the perceptual salience of morphological constituents within the word may bias reading toward morphological decomposition. In this vein, the focus of the present paper is to examine whether also a formal property of the root, such as its length, influences word processing. To the best of our knowledge, up until now the effect of root length on reading speed and accuracy has been considered only in the study by Hyönä and Pollatsek (1998) who included length of the first morphemic constituent among the predictors of the pattern of adult readers’ eye-movements. It is well known from eye-tracking studies that, at least for adult readers, longer morphologically complex words are more subject to morphemic decomposition than shorter ones, with increasing word length enhancing the probability of morphological processing (see, among others, Bertram and Hyönä, 2003; Niswander-Klement and Pollatsek, 2006). Hyönä and Pollatsek (1998) went on and assessed whether also the length of the first component had an influence on the locations of fixations in reading compound words, based on the idea that the morphemes in a word could guide eye movements just as words do (see also, more recently, Hyönä et al., 2018) and that the visual width of the morphemic constituent could control the size of the saccade (see also Kuperman et al., 2010).

In particular, Hyönä and Pollatsek (1998) anticipated a difference in landing position when a word included either a short or a long first morphemic constituent, with eye fixations being farther into a word the longer the initial morpheme. In Hyönä and Pollatsek’s (1998) study the length of the initial morpheme influenced the location of the second fixation on the target word and the patterns of re-fixations and fixation durations: The second fixation was farther in the word when first morphemes were longer. There were more intra-word regressions when the first morpheme was short then when it was long. When the first morpheme was long, the first-fixation duration was shorter but the second-fixation duration was longer. Thus, the pattern of eye movements appeared to be at least partly guided by processing of the morphemic components of the word.

However, it also appeared that first-morpheme length was not controlling the subsequent eye-movement when the initial fixation was near the beginning of the word. When the initial fixation landed on the first four letters of the word, there was only marginal control of eye movements by morphemes. By contrast, when the initial fixation landed near the middle of the word, the length of the initial morpheme had an influence on the length of the initial fixation, and there was a greater modulation of the location of the second fixation. This is consistent with data reported for the reading of isolated words by O’Regan et al. (1984) and Vitu et al. (1990), who found that, when the initial fixation occurred in a “bad” location (i.e., far from the optimal viewing position), a corrective eye movement was made to a more advantageous viewing location (presumably nearer the middle of the word). In summary, in the study of Hyönä and Pollatsek (1998) morphemic processing was more complete when the initial fixation was nearer the middle of the word (and in this case, the role of root length emerged). When the initial fixation was near the beginning of the word, guidance of the fixation appeared largely affected by oculomotor factors. According to the authors, these differences indicate that not all eye-movement behavior is guided by morphemic processing, but there is a compromise between visual and morphemic guidance, which is likely to be acquired during reading development.

It is well known that both the effects of visuo-perceptual and linguistic factors on the recognition of words and the viewing-position effects are modulated by print exposure and reading limitations (Ducrot et al., 2013). By fifth-sixth grade, the size of the visual span of typically developing children and most of the indices of their eye movements during reading are already very much like those of adults (Rayner, 1986; Kwon et al., 2007; Häikiö et al., 2009). By contrast, the visuo-attentional span of same age children with dyslexia is smaller than that of skilled readers (Bosse et al., 2007; Bosse and Valdois, 2009). The eye movements of dysfluent readers as old as 16–36 years still reveal a deficiency in the early serial orthographic processing of those words that do not have solid orthographic memory representations (Hautala and Parviainen, 2014).

It can consequently be envisaged that the modulation of the reading behavior induced by morphemic constituents of different sizes requires a flexible reading system, which, however, may be deficient in children with dyslexia. On the basis of the evidence on adult readers (Hyönä and Pollatsek, 1998), we thought that the length of the root could have a differential role in the reading behavior of children with and without dyslexia, respectively. In order to be processed as a unit in a single fixation, longer roots may require a mature level of visuo-perceptual integration, as the one already present in typical readers of sixth grade. For these children, we expected that the wider is the perceptual unit corresponding to the root (i.e., the longer is the root), the higher is the advantage that a reader can gain from the recognition of the root morpheme over the decoding of the long low-frequency word. Take, for example, two Italian suffixed words like ‘nasino’ (small nose) and ‘cavallino’ (young horse) that differ in root length (‘nas-’ and ‘cavall-,’ three- and six-letter long, respectively). Our prediction is that the recognition of a longer root like ‘cavall-’ should produce more advantage in reading the long and low-frequency word ‘cavallino’ than a shorter root like ‘nas-’ does in reading the word ‘nasino.’

We further hypothesized that the visuo-perceptual limitations of children with dyslexia made it more likely that their initial fixation occurred near the beginning of the word (Hawelka et al., 2010).^1^ This eye-movement behavior should allow children with dyslexia to fully process shorter roots but might result in frequently missing the middle of longer words, which is required for full processing of longer roots.

Although our main hypothesis assigns a leading role to the root in affecting reading speed, effects of suffix properties cannot be excluded. Indeed, there is evidence of the role of derivational suffix knowledge in decoding morphologically complex words (e.g., see Mann and Singson, 2003). Furthermore, several studies have shown effects of suffix properties in lexical decision tasks and on the eye-movement behavior of children (e.g., Lázaro et al., 2017) and adults (e.g., Ford et al., 2010; Kuperman et al., 2010). In previous studies on Italian children, both with and without dyslexia, although not affecting reading speed, the presence of a suffix in a pseudoword exerted a facilitating effect on reading accuracy (Traficante et al., 2011). However, the relative contributions of root and suffix properties on the reading aloud of words has not yet been assessed.

Overall, the present study assessed the role of root length on the performance of children with and without developmental dyslexia in reading derived words by means of an experimental regression design in which, along with root length, several other predictors related to word, root and suffix properties, were included. We focussed on low frequency derived words given the evidence discussed above for morphemic effects particularly on these forms. We expected that all children would benefit from morphological processing in terms of reading speed, with faster reading times for words embedding higher-frequency roots. The frequency of the suffix was not expected to have a particular role on reading times, at least not on those of children with dyslexia, given their visuo-perceptual limitations and their serial scanning procedure. The length of the root was expected to positively modulate the reading speed of typically developing readers only, with larger facilitating effects driven by longer than shorter roots. However, the length of the root was not expected to influence the reading times of children with dyslexia because of their visuo-perceptual limitations in processing long stimuli.

## Materials and Methods

### Participants

Twenty children with dyslexia participated to the study: eight children were examined at the Centre for Cognitive and Linguistic Disorders (ASL 1) in Rome and 12 children were selected during a screening carried out in 6th grade classes of junior high-schools in Milan. All children showed a marked reading delay in two standardized tests: text reading (*MT Reading test*; Cornoldi and Colpo, 1998), and word list reading (*Word Reading* subtest from the *Developmental Dyslexia and Dysorthography battery*; Sartori et al., 1995). Time (in sec/syllable) and accuracy (number of errors) were measured. Raw scores were converted to *z*-scores according to Italian normative data. All children with dyslexia scored at least 1.65 *z*-scores below the normative values for reading speed and/or below the fifth percentile for accuracy in at least one of the measures.

Readers with dyslexia were compared to 40 typically developing children of the same chronological age, assessed in 6th grade classes of junior high-schools in Milan (*N* = 28) and Rome (*N* = 12). Performances on the *MT Reading test* and on the *Word Reading* subtest were well within normal limits for both reading speed and accuracy. As a group, readers with dyslexia were slower than controls in the *MT Reading test* by 76% and in the *Word Reading* sub-test by 81%, respectively. Summary statistics and mean scores on the screening tests are presented in **Table 1**.

**Table 1:** Means (and standard deviations in parentheses) for age, performances on the *Raven Test*, on text passage reading from the *MT Reading Test* and Reading of words sub-test from the *Developmental Dyslexia and Dysorthography Battery*.

	Children with dyslexia		Typically developing children	
		
Age – in months	141.50 (4.3)		140.57 (4.5)	
	**Raw score**	***z* score/ percentile**	**Raw score**	***z* score/percentile**
Raven test – correct responses	29.42 (3.5)	-	30.54 (3.7)	–
Time (Text passage) – sec/syllable	0.44 (0.07)	-1.81 (0.78)	0.26 (0.03)	0.13 (0.38)
Accuracy (Text passage) – no of errors	22.0 (17.3)	-1.95 (2.4)	7.7 (4.1)	0.04 (0.57)
Time (Word Reading) – sec/word	1.18 (0.24)	-1.47 (0.8)	0.65 (0.10)	0.30 (0.3)
Accuracy (Word reading)° - no of errors	8.5 (5–32)	20^∗^	1 0-4	0^∗^


*Data are separately presented for children with dyslexia and typically developing children. °Medians (and ranges) are reported for this parameter. ^∗^Number of children with a score at or below the fifth percentile.*

The two groups of readers were matched for gender (5 girls and 15 boys in the group of children with dyslexia; 11 girls and 29 boys in the group of skilled children), age, and non-verbal intelligence (*Raven’s Colored Progressive Matrices*; Italian adaptation, Pruneti et al., 1996; see **Table 1**). All children had normal or corrected-to-normal vision.

The study was carried out according to the principles of the 2012–2013 Helsinki Declaration. Written informed consent to participate in the study was obtained from the parents of all children. The study was approved by the IRB of the Department of Psychology of Sapienza University of Rome.

### Materials

Sixty low-frequency derived words were selected, composed of a root and a derivational suffix (e.g., PIED-INO, ‘little foot’). All words were orthographically, phonologically and semantically transparent with respect to their base word, and included familiar roots and suffixes. Word frequency, root frequency, root family size, and suffix frequency were calculated on a written child frequency count (*Elementary lexicon: Statistical data on written and read Italian language in primary school children*; Marconi et al., 1993). Descriptive statistics for the experimental variables are reported in **Table 2**. The complete list of the experimental stimuli is available in the Supplementary Material.

**Table 2:** Descriptive statistics for the psycholinguistic features of the experimental stimuli (*N* = 60).

	*M*	*SD*	Minimum	Maximum
Word frequency	16.55	17.3	0	56
Word length	8.38	1.4	6	11
Root frequency	618.57	714.9	83	3676
Root family size	3.28	2.0	1	11
Root length	4.50	1.1	3	6
Suffix frequency	766.43	462.4	15	2147
Suffix length	3.72	0.6	3	5


*Word length, in letters; Root frequency, sum of all word tokens that share the root; Root family size, number of all different word types sharing the root; Root length, in letters; Suffix frequency, sum of all word tokens ending with the suffix; Suffix length, in letters. All the measures of frequency and family size are calculated on 1 million occurrences in a written child frequency count (Marconi et al., 1993).*

Sixty simple words, matched to the derived words for length and word frequency were added as fillers to the list of experimental stimuli, for a total of 120 word stimuli. The inclusion of simple word fillers aimed at preventing the induction of a forced parsing strategy, which could be present had the list included only morphologically complex words. All words had the most frequent Italian stress pattern, on the penultimate syllable.

### Procedure

The stimuli were presented in black lower case (font Courier New 18pt bold) in the center of the computer screen. Each stimulus was preceded by a fixation point (300 ms), followed by a brief interval (250 ms). Each word remained on the screen until the onset of pronunciation, or for a maximum of 6000 ms. There was an inter-stimulus interval of 1400 ms.

The 120 test items were presented in four blocks of 30 trials each. Order of presentation was randomized both within and between blocks. A short pause followed each block. Before the first experimental block the participants completed a practice block, consisting of 10 items with similar characteristics as the experimental items, presented in random order.

Participants were instructed to read aloud the words that appeared on the computer screen as fast and accurately as possible. The children were tested individually in a quiet room at school or at the clinical center. Responses were recorded by a microphone connected to a voice-key. Performance in terms of RTs was measured in ms using the E-Prime software. The experimenter manually noted mispronunciation errors.

### Data Analysis

Invalid trials due to technical failures accounted for 3.9 and 1.6%, in children with dyslexia and in typically developing readers, respectively, and were treated as missing data. Pronunciation errors were excluded from the analyses on RTs.

As expected, children with dyslexia were much slower (*M* = 1475 ms, *SD* = 464.6, range: 850–2342 ms) than typically developing children (*M* = 701 ms, *SD* = 130, range: 468–985). Children with dyslexia also made more reading errors (9.6%, range: 0–30%) than typically developing children (2.2%, range: 0–18%). The two groups of readers were quite different in both RTs (*t* = 7.3, *df* = 58, *p* < 0.001) and accuracy (*t* = -4.52, *df* = 58, *p* < 0.001). Thus, because of the large difference between groups both in mean values and in dispersion measures, the two groups were considered as two separate statistic populations and analyses of data were carried out within each group separately.

Log-transformed RTs and accuracy of responses in binary form (Correct = 1, Error = 0) were considered as dependent variables. Mixed-effects regression models (Baayen, 2008) were carried out, with participants and items as random intercepts, and six fixed effect predictors: word frequency, word length, root frequency, root family size, root length, and suffix frequency. Suffix length was not considered because it was linearly dependent on root length. Pearson’s correlations across items were calculated on a by-item basis to determine how word features were related within stimuli (**Table 3**). All correlations between experimental variables except one (see below) were well below the 0.60 threshold (indeed all < 0.30), ensuring that there were no critical multicollinearity concerns (Tabachnick and Fidell, 2007). Root family size was moderately but significantly correlated with both word frequency (*r* = 0.27) and root frequency (*r* = 0.28). Because of the high correlation between word length and root length (*r* = 0.79), a residualization process was applied, in which root length was predicted from word length. The unexplained residuals from this regression analysis were included in the mixed-effects models instead of raw root length. To reduce skewness of the distributions and decrease the influence of atypical outliers, word frequency, root frequency and suffix frequency were logarithmically transformed, whereas word length and root family size were standardized (see Kuperman et al., 2010).

**Table 3:** Pearson’s correlation indices among raw variables.

	Word frequency	Word length	Root frequency	Family size	Root length
Word frequency	-				
Word length	0.057	-			
Root frequency	0.184	-0.187	-		
Family Size	0.275^∗^	-0.128	0.279^∗^	-	
Root length	0.073	0.787^∗∗∗^	-0.167	-0.134	-
Suffix frequency	0.039	-0.131	0.066	-0.224	-0.010


*^∗^*p* < 0.05, ^∗∗∗^*p* < 0.001.*

Different mixed-effects regression models with variables referred to whole word (word frequency and word length), root (root frequency, root family size, root length), and suffix frequency, respectively, were refitted through the model criticism procedure (Baayen, 2008). Models were compared using Akaike Information Criterion (AIC): AIC = 2k – 2ln(L), where k = number of parameters and L = maximum likelihood. Each (*N*) model was derived from the previous (*N-1*) model after removing non-significant effects. The model with all variables reaching significance level and associated to the numerically lowest AIC was considered the best model fitting data. Analyses were carried out by means of the statistical software R (R Development Core Team, 2009), using lme4 package (Bates et al., 2015).

For accuracy, generalized mixed-effects regression models with Laplace’s approximation for binomial data were carried out.

## Results

### Typically Developing Children

The AIC index identified Model 6, representing the linear combination of word length, root length, root frequency, and family size, as the best model fitting RT data (**Table 4**). In this model, none of the interactions tested in the other models (indicated by the ‘^∗^’ symbol) reached significance level.

**Table 4:** Typically developing children: Comparison of mixed-effects regression models on RTs (best fitting model in bold).

Model	AIC
Model 1 - Word length ^∗^ Word frequency + Root length^∗^Root frequency^∗^Root family Size + Suffix frequency + (1| subject) + (1| items)	-403.03
Model 2 - Word length ^∗^ Word frequency + Root length^∗^Root frequency + Root family Size + Suffix frequency + (1| subject) + (1| items)	-406.05
Model 3 - Word length + Word frequency + Root length^∗^Root frequency + Root family Size + Suffix frequency + (1| subject) + (1| items)	-408.04
Model 4 - Word length + Word frequency + Root length + Root frequency + Root family Size + Suffix frequency + (1| subject) + (1| items)	-408.3
Model 5 - Word length + Word frequency + Root length + Root frequency + Suffix frequency + (1| subject) + (1| items)	-410.2
**Model 6 - Word length + Root length + Root frequency + Suffix frequency + (1| subject) + (1| items)**	**-410.7**
Model 7 - Word length + Root length + Root frequency + (1| subject) + (1| items)	-408.64
Model 8 - Word length + Root length + Suffix frequency + (1| subject) + (1| items)	-403.37
Model 9 - Word length + Root frequency + Suffix frequency + (1| subject) + (1| items)	-408.46
Model 10 - Root length + Root frequency + Suffix frequency + (1| subject) + (1| items)	-402.84


Coefficients of the best mixed-effects regression model selected are presented in **Table 5**. The model showed that word length and root length had opposite effects on latencies. The longer the word, the slower was the response (*b* = 0.03, *t* = 3.98, *p* < 0.001). However, the effect of root length was in the opposite direction (*b* = -0.01, *t* = -2.02, *p* = 0.047), as increases in (the residual values of) root length were associated to faster RTs. The effect of root frequency was significant (*b* = -0.02, *t* = -2.71, *p* = 0.008), indicating that the higher was the frequency of the root, the faster was the response. Suffix frequency had a significant effect (*b* = -0.02, *t* = -2.22, *p* = 0.03): the higher the frequency of the suffix, the faster the response.

**Table 5:** Typically developing children: Coefficients of the best mixed-effects model on RTs.

*Random effects*	*SD*				

Participant	0.1639				
Item	0.0476				
Residual	0.1478				

***Fixed effects***	***Estimate***	***t-*value**	**Pr ( > |*t*| )**	***F*-value**	**Pr ( > *F*)**

(Intercept)	6.7478	88.227	<0.001		
Word length	0.0277	3.977	<0.001	9.1023	0.004
Root length	-0.0140	-2.022	0.047	4.0697	0.047
Root frequency	-0.0226	-2.708	0.008	9.9391	0.003
Suffix frequency	-0.0174	-2.220	0.03	3.6881	0.06


For accuracy, the AIC index identified Model 4, representing the linear combination of word frequency and suffix frequency, as the best model (**Table 6**).

**Table 6:** Typically developing children: Comparison of generalized mixed-effects regression models on accuracy (best fitting model in bold).

Model	AIC
Model 1 - Word length + Word frequency + Root length + Root frequency + Suffix frequency + (1| subject) + (1| items)	493
Model 2 - Word length + Word frequency + Root length + Suffix frequency + (1| subject) + (1| items)	491.5
Model 3 - Word frequency + Root length + Suffix frequency + (1| subject) + (1| items)	489.6
**Model 4 - Word frequency + Suffix frequency + (1| subject) + (1| items)**	**488.4**
Model 5 - Word frequency + (1| subject) + (1| items)	492.8
Model 6 - Suffix frequency + (1| subject) + (1| items)	490.4


Both word frequency (*b* = 0.22, *z* = 2.03, *p* = 0.043) and suffix frequency (*b* = 0.41, *z* = 2.69, *p* = 0.007) were significant: accuracy was higher for higher-frequency words and when the word included a frequent suffix.

### Children With Dyslexia

The AIC index identified Model 7, representing the linear combination of word length, root frequency, and family size, as the best model fitting RT data (**Table 7**). In this model, none of the interactions tested in the other models (indicated by the ‘^∗^’ symbol) reached significance level.

**Table 7:** Children with dyslexia: Comparison of mixed-effects regression models on RTs (best fitting model in bold).

Model	AIC
Model 1 - Word length ^∗^ Word frequency + Root length^∗^Root frequency^∗^Root family Size + Suffix frequency + (1| subject) + (1| items)	905.31


Model 2 - Word length ^∗^ Word frequency + Root length^∗^Root frequency + Root family Size + Suffix frequency + (1| subject) + (1| items)	902.14


Model 3 - Word length ^∗^ Word frequency + Root length^∗^Root frequency + Root family Size + (1| subject) + (1| items)	902.19


Model 4 - Word length + Word frequency + Root length^∗^Root frequency + Root family Size + (1| subject) + (1| items)	901.18


Model 5 - Word length + Root length^∗^Root frequency + Root family Size + (1| subject) + (1| items)	901.58


Model 6 - Word length + Root length + Root frequency + Root family Size + (1| subject) + (1| items)	902.73


**Model 7 – Word length + Root frequency + Root family Size + (1| subject) + (1| items)**	**900.9**


Model 8 - Word length + Root frequency + (1| subject) + (1| items)	902.92


Model 9 - Word length + Root family Size + (1| subject) + (1| items)	910.89


Model 10 - Root frequency + Root family Size + (1| subject) + (1| items)	906.19




**Table 8** shows the main effects that reached significance level. A word length effect was observed, such that the longer was the word, the slower was the response (*b* = 0.05, *t* = 3.62, *p* < 0.001). Also in children with dyslexia root frequency had a facilitating effect (*b* = -0.05, *t* = -3.67, *p* < 0.001): the higher the frequency of the root, the faster the response. A negative effect of root family size (*b* = 0.03, *t* = 2.22, *p* = 0.03) emerged in children with dyslexia: the larger the root family size, the longer children’s RTs. Differently from typically developing readers, children with dyslexia did not show any effect of root length.

**Table 8:** Children with dyslexia: Coefficients of the best mixed-effects model on RTs.

*Random effects*	*SD*				

Participant	0.2963				
Item	0.0609				
Residual	0.3217				

***Fixed effects***	***Estimate***	***t-*value**	**Pr (>|*t*| )**	***F*-value**	**Pr (>*F*)**

(Intercept)	7.5393	61.960	<0.001		
Word length	0.0465	3.620	0.0006	7.3208	0.009
Root frequency	-0.0485	-3.675	0.0005	12.3326	0.0009
Root family size	0.0320	2.217	0.03	3.5890	0.06


For accuracy, the AIC index identified Model 3, representing the linear combination of word frequency and suffix frequency, as the best model (**Table 9**).

**Table 9:** Children with dyslexia: Comparison of generalized mixed-effects regression models on accuracy (best fitting model in bold).

Model	AIC
Model 1 - Word length + Word frequency + Root length + Suffix frequency + (1| subject) + (1| items)	711.5


Model 2 - Word frequency + Root length + Suffix frequency + (1| subject) + (1| items)	710.2


**Model 3 - Word frequency + Suffix frequency + (1| subject) + (1| items)**	**709**
Model 4 - Word frequency + (1| subject) + (1| items)	718.3
Model 5 - Suffix frequency + (1| subject) + (1| items)	710.6


Similar to typically developing peers, generalized mixed-effects regression models on accuracy data showed the main effects of word frequency (*b* = 0.14, *z* = 1.93, *p* = 0.054) and of suffix frequency (*b* = 0.43, *z* = 3.64, *p* < 0.001): pronunciation accuracy was higher for more frequent words and when the word included a frequent suffix.

## Discussion

The reading aloud of children, both with and without dyslexia, is usually facilitated by the possibility of parsing a long word into its constituent morphemes, roots and affixes (Burani, 2010). The present study focussed on the possible effects of a visuo-formal property of the root (i.e., root length) in affecting morphological parsing during children’s reading of low-frequency suffixed derived words. The results of the present experiment confirmed the expectation that, in typically developing children attending sixth grade, the presence of a longer root fosters morphemic access with consequent faster reading latencies. Long roots were not expected to result in a greater reading benefit over shorter roots in children with dyslexia because of their limitations in visuo-perceptual processing and in their eye movements’ behavior. The experimental data confirmed also this expectation.

Notably, several other predictors also exerted some effects on children’s reading latencies to the suffixed derived words. For both typically developing readers and children with dyslexia, the main effect of word length indicated faster RTs for shorter than longer words. A general effect of word length may be expected due to the characteristics of the stimuli used, i.e., long low-frequency words. The main effect of root frequency indicated faster RTs for words including more frequent roots. Neither for skilled readers nor for children with dyslexia, whole-word frequency played a significant role on RTs to low-frequency derived words. The lack of significance for word frequency is in keeping with the idea of a massive use of morphemic parsing in reading aloud low-frequency long words in both groups. At the same time, the large root frequency effect for both groups indicates that the root was accessed as the main reading unit, irrespective of reading ability. The difficulty in using the whole word as an access unit for these long low-frequency words was also suggested by the inhibitory effect of word length for both groups of children.

The effect of root length was different in the two groups. In the case of typically developing children, both word and root length influenced reading times, but in opposite directions: word length negatively affected reading latencies while root length positively affected reading latencies, with longer roots leading to shorter latencies. It is thus confirmed that, for skilled readers, root length positively affects naming times over and above the opposite (and potentially confounding) effect of word length. This advantage may indicate that the longer is the morphemic constituent, the more informative is the reading unit that can be identified in the string of letters, thus favoring reading fluency. Overall, the effectiveness of longer roots on reading latencies could be caused by the combined effects of their perceptual salience within the word, and the fact that they are particularly informative access reading units. This may be because they have less lexical competitors (i.e., fewer competing root neighbors) than shorter roots (Marian et al., 2012). It might be interesting in further research to jointly examine the influence of root length and root neighborhood. For children with dyslexia the selective influence of root length found in typically developing children was absent, suggesting that their visuo-perceptual limitations may limit the possibility of a long root to exert its positive effect on reading. It should be added that the different pattern of results in children with dyslexia and typically developing readers was obtained through separate analyses (due to their basic large differences in speed). Accordingly, it would certainly be important to replicate these findings on separate samples. Possibly, if the two groups of children are not so different in terms of reading skills this might also allow running a single analysis which would certainly be informative.

The present results indicate that not only the frequency of the root but also its length is an important parameter in modulating morphological processing. It should be noted that the existing computational models of reading aloud in Italian (i.e., Pagliuca and Monaghan, 2010; Perry et al., 2014) both have the potentiality to account for our findings. Both models have been developed for capturing the statistical regularities of the spelling-to-sound mapping in Italian polysyllabic words and both have shown sensitivity to large grain sizes in reading Italian. Both the CDP++ (Perry et al., 2014) and the model of Pagliuca and Monaghan (2010) were successful in simulating morphological effects in reading aloud Italian polysyllabic pseudowords even though they do not have either explicit morphological processing layers or semantics, consistently with the idea that morphological effects in word naming are non-semantic in nature (Burani et al., 1999). Thus, if these models would also prove adequate to capture the effects found in the present study, this would suggest that these effects can be explained by factors correlated with morphemic status, such as frequency or size of the chunks corresponding to morphemes, rather than some sort of explicit morphological status or the semantics associated with particular morphemes. If, by contrast, in simulating our results on word reading the differences between the two models (i.e., the absence of lexical units in the model by Pagliuca and Monaghan (2010), and the presence of these units in the CDP++ model) should result in a better performance of one of the models over the other, this would help adjudicating on the lexical vs. non-lexical status of morphemes as reading units.

Two less expected results were found in typically developing children and in children with dyslexia, respectively. A facilitating effect of suffix frequency was found on typically developing children only: words including more frequent suffixes were read faster than words with less frequent suffixes. A negative effect of root family size was found on dyslexics’ reading speed: words that included a root which is present in several different words were read aloud slower than words including a root occurring in fewer different words.

Both these findings may have their source in the different reading scanning procedures adopted by the two populations of readers. The results of the present study confirmed that in the presence of long low-frequency words composed of morphemes, both typically developing children and children with dyslexia rely on the root as the main access unit driving morphemic parsing and latencies. However, skilled readers may extend their processing farther in the word at the extent that their reading latencies are speeded up by the presence of a suffix frequently occurring at the end of a complex word. By contrast, children with dyslexia may not be able to exploit the word ending information to accelerate their vocal RTs. This interpretation is consistent with several findings in the literature that show that less skilled readers use parafoveal information less effectively than do more skilled readers. Unlike faster readers, slower readers focus their attention more on foveal processing during a fixation (e.g., Häikiö et al., 2009) and are delayed in detecting word-end information (Hautala and Parviainen, 2014). Overall, increasing reading proficiency involves the ability to more effectively use partial word information available in parafoveal vision (Rayner, 1986). Thus, our skilled young readers may have started pronunciation on the basis of information coming from both the foveally fixated root and the parafoveally processed suffix.

A similar reasoning may also account for the negative effect of root family size found on the reading times of children with dyslexia. Words with a root that occurs in many different word-types are more easily recognized and have faster lexical access in lexical decision in both adults (e.g., see Schreuder and Baayen, 1997; Bertram et al., 2000) and children (Perdijk et al., 2012). However, similarly to our skilled children, a large morphological family did not affect word reading aloud in adults (Baayen et al., 2006, 2007). According to the latter authors, the family size facilitating effect is driven by the property that different words sharing the root also share their semantics, i.e., they share part of their meanings. For this reason, family size effects would occur in a task that implies access to semantics, like lexical decision, but would not be found in a task like fast reading aloud that is largely impermeable to the semantic properties of the words (Burani et al., 1999; Baayen et al., 2007).

The reversed family size effect found in children with dyslexia might be due to a different reason, namely to their characteristic serial processing in reading. Let us consider two examples of words differing in root family size that were presented to our participants: the first word, ‘autista’ (driver) has a root (‘aut-’) with a large family size (‘auto’ - car, ‘autobus’ - bus, ‘autocarro’ - van, ‘automobile’ - car, ‘automobilista’ - driver, ‘autoscontro’ - dodgem, ‘autostrada’ - highway), whereas the second word, ‘durezza’ (hardness) has a root (‘dur-’) which only occurs in another word in the child corpus (‘duro,’ hard). For readers whose reading limitations make it unlikely that the suffix is taken into account when starting pronunciation, a word like ‘durezza,’ whose root is present only in two words, might lead to less uncertainty in planning pronunciation of the whole word, while a word like ‘autista,’ which includes a root compatible with several possible different words, may delay the start of pronunciation. A similar effect has been reported by Traficante et al. (2014), who observed that children had higher error rates when the root of a derived word was compatible with several alternatives. The pattern of effects we found for root family size may appear in contrast to the effects reported by Carlisle and Katz (2006), cited in the Section “Introduction,” who found that fourth and sixth English speaking graders read derived words with large morphological families more accurately than words with small families. However, the word sets used by Carlisle and Katz (2006) also differed in base word frequency, which was higher for words with a large family size than for words with a small family size. Crucially, Carlisle and Katz did not use a regression design that allowed to evaluate whether the effect of family size on derived word reading survived controls for correlated properties such as base frequency or cumulative frequency of the words present in the family (our root frequency). Therefore, the facilitating family size effect they found could be driven by the high root frequency of words with a large family size (see also Deacon and Francis, 2017, for empirical demonstration that only base frequency made an independent contribution to reading accuracy of English-speaking children in Grades 3 and 5 beyond the influence of the control variables base family size and family frequency).

Although not being the focus of the present research, our results concerning reading accuracy deserve some comment. For both typically developing readers and children with dyslexia pronunciation accuracy was positively affected by word and suffix frequency. In order to interpret these effects, it should be considered that pronunciation accuracy reflects the part of reading processing that takes place after the onset of pronunciation. In order to start pronunciation, articulatory planning of the whole word is not necessary, but the reader can start reading aloud on the basis of a sub-part of the whole word (Rastle et al., 2000; Sulpizio et al., 2015), i.e., in the present case, on the basis of the initial morpheme (i.e., the root; Bertram and Hyönä, 2003). Then, after starting pronunciation, the pronunciation of the whole stimulus must be correctly completed. To this end, several properties of the word and its constituent parts may play a role. The frequency of occurrence of the whole stimulus, which reflects repeated exposures to a given word, is one of these properties. However, as Baayen (2010) has shown, in addition to the amount of exposure to the word, part of the word frequency effect in a morphologically complex word also reflects the probability that a given root is followed by a given suffix: thus, the higher this probability (i.e., the higher the probability of the combination of that root with that specific suffix), the more accurate its production can be. Both sources of information (i.e., the total amount of exposure to the whole word, and the word’s local morphological micro-context or co-occurrence) affect the probability that a given word is assigned a correct pronunciation.

The second variable that affected correctness of pronunciation of both groups of children was suffix frequency, with higher accuracy driven by higher-frequency suffixes. This finding can be seen as the complementary effect of the root frequency effect on latencies: for all children, the frequency of the first constituent affects the pronunciation onset, but the correctness of the pronunciation of the whole string depends on the recognition of the ending perceptual chunk, i.e., the suffix. The effect of suffix frequency on the reading accuracy of children, and of children with dyslexia in particular, must have its source on some properties of suffixes in driving pronunciation after starting the pronunciation process. It has been often shown in developmental reading studies that suffixes act as stress attractors (e.g., Jarmulowicz et al., 2007, 2008) and more generally provide a cue to stress position, i.e., to where stress should be placed when pronouncing a word (Grimani and Protopapas, 2017). This is crucial in pronouncing polysyllabic words like Italian derived words, which are usually 3-to-5 syllables long. The role of suffixes in cueing stress position is particularly important if we consider that children start pronunciation of morphologically complex words by planning pronunciation of the root alone. For most derived words (and for all the words we considered in our study), following root parsing, the root’s lexical stress must be shifted forward in the word. For example, for a word like ‘gattino’ (kitten) pronunciation may start by planning the root (‘gatt-,’ cat) pronunciation, whose stress is ‘gAtt-’. However, assembling the pronunciation of the root with that of the suffix after parsing entails re-assigning stress to the complex word, so that the correct stress (i.e., ‘gattIno’) is finally assigned. In such a complex process, the more frequent the suffix, the easier is stress assignment and the consequent co-articulation of the root-suffix combination. The present findings confirm those of Traficante et al. (2011) who showed that the presence of a suffix in a pseudoword did not influence the onset of children’s pronunciation but had a role in enhancing pronunciation accuracy.

In summary, the results of the present experiment confirm that, when reading low-frequency suffixed derived words, both typically developing children and children with dyslexia largely rely on morphemes as reading units, and especially on the root as providing a head-start to pronunciation. The novel finding of this study is that, for skilled 6th grade readers, the longer is the root, i.e., the wider is the chunk that can be recognized at the beginning of the word, the more it can speed up the onset of stimulus pronunciation. By contrast, readers with dyslexia show a benefit from accessing the root, that is not modulated by its length. Longer roots do not result in particular benefits for readers with dyslexia as compared to shorter roots, probably because they are more likely to exceed the width of their visual scanning. These findings need to be accommodated within a model of morphological processing which accounts for the respective roles of visuo-perceptual properties of morphemes, such as root length, and differing reading abilities.

## Author Contributions

All authors listed have made a substantial, direct and intellectual contribution to the work, and approved it for publication.

## Conflict of Interest Statement

The authors declare that the research was conducted in the absence of any commercial or financial relationships that could be construed as a potential conflict of interest. The reviewer CM and handling Editor declared their shared affiliation.

## Footnotes

1 Note that perceptual limitations are selective for orthographic materials and children with dyslexia are not generally impaired in oculo-motor mechanisms *per se*; in particular, they show a spared mechanism for correcting fixations in suboptimal landing positions (Gagl et al., 2014).
