# Supplementary material for: Reading Derived Words by Italian Children With and Without Dyslexia: The Effect of Root Length
Source: Front Psychol. 2018 May 8;9:647. doi: 10.3389/fpsyg.2018.00647 (PMC5952107; doi:10.3389/fpsyg.2018.00647)
Supplement: Supplementary file 1 [file Table_1.docx]

APPENDIX.

*Experimental derived words.*

| *acquario* | *aquarium* |
| --- | --- |
| *autista* | *driver* |
| *bambolotto* | *doll* |
| *calciatore* | *football player* |
| *casetta* | *small house* |
| *cavallino* | *young horse* |
| *conoscenza* | *knowledge* |
| *corsetta* | *jog* |
| *ditone* | *big toe* |
| *divertimento* | *entertainment* |
| *dolcetto* | *pastry* |
| *durezza* | *hardness* |
| *entrata* | *entrance* |
| *esistenza* | *existence* |
| *finale* | *final* |
| *fioraio* | *florist* |
| *foglietto* | *small piece of paper* |
| *fumatore* | *smoker* |
| *gattino* | *kitten* |
| *gelataio* | *ice-cream vendor* |
| *giovanotto* | *young man* |
| *giretto* | *stroll* |
| *grandezza* | *greatness* |
| *guerriero* | *warrior* |
| *importanza* | *importance* |
| *libraio* | *bookseller* |
| *lontananza* | *remoteness* |
| *maglietta* | *t-shirt* |
| *mancanza* | *lack* |
| *mattinata* | *morning* |
| *muratore* | *bricklayer* |
| *nasino* | *little nose* |
| *negoziante* | *shopkeeper* |
| *nipotino* | *grandbaby* |
| *occhiata* | *glance* |
| *ossario* | *ossuary* |
| *osservatore* | *observer* |
| *pagamento* | *payment* |
| *parolaccia* | *dirty word* |
| *partenza* | *departure* |
| *pastorello* | *little shepherd* |
| *piedino* | *little foot* |
| *potenza* | *power* |
| *ragazzino* | *little boy* |
| *regalino* | *little present* |
| *rendimento* | *profit* |
| *salvezza* | *safety* |
| *scarpone* | *boot* |
| *scherzetto* | *joke* |
| *sedile* | *seat* |
| *serata* | *evening* |
| *servitore* | *servant* |
| *sorellina* | *little sister* |
| *stellina* | *little star* |
| *storiella* | *short story* |
| *trattamento* | *treatment* |
| *vecchiaia* | *old age* |
| *villetta* | *cottage* |
| *visiera* | *visor* |
| *zampina* | *small leg* |
